# Ga-doped Ca_12_Al_14_O_33_ mayenite oxide ion conductors: synthesis, defects, and electrical properties†

**DOI:** 10.1039/c8ra08254e

**Published:** 2019-01-30

**Authors:** Huaibo Yi, Yun Lv, Yanhui Wang, Xue Fang, Victoria Mattick, Jungu Xu

**Affiliations:** MOE Key Laboratory of New Processing Technology for Nonferrous Metals and Materials, Guangxi Universities Key Laboratory of Non-ferrous Metal Oxide Electronic Functional Materials and Devices, College of Materials Science and Engineering, Guilin University of Technology Guilin 541004 P. R. China xujungu@glut.edu.cn; Department of Chemical Engineering, University of South Carolina Columbia SC 29201 USA

## Abstract

Although mayenite Ca_12_Al_14_O_33_ has been known as an oxygen ion conductor for several decades, its relatively low oxide ion conductivity limits its applications in electrochemical devices. Thus, many efforts have been made by researchers, employing a doping strategy, in order to further improve its ionic conductivity, but with little success. In this work, a series of pure phase Ca_12_Al_14−*x*_Ga_*x*_O_33+*δ*_ (0 ≤ *x* ≤ 1.2) materials were synthesized by a traditional solid state reaction method. Scanning electron microscopy (SEM) combined with energy dispersion spectrum (EDS) analyses disclosed well-sintered ceramics with uniform Ga distributions. The defect formation energies for Ga replacing the two distinguishable Al1 and Al2 sites in Ca_12_Al_14_O_33_ calculated by static lattice atomistic simulation are nearly identical, ∼3.03 and ∼3.04 eV, respectively, consistent with the results of Rietveld refinements based on the XRD data, from which no preferred distribution of Ga on Al1 or Al2 site was observed. The electrical properties investigated by alternating current (AC) impedance spectroscopy show increased bulk conductivities for 0 ≤ *x* ≤ 0.4. Thus, here we present the first work that successfully improves the bulk oxide ion conductivity of Ca_12_Al_14_O_33_ by Ga-doping.

## Abstract 2

The bulk conductivity of Ca_12_Al_14_O_33_ can be apparently enhanced by Ga-doping on the Al sites.

## Graphical abstract

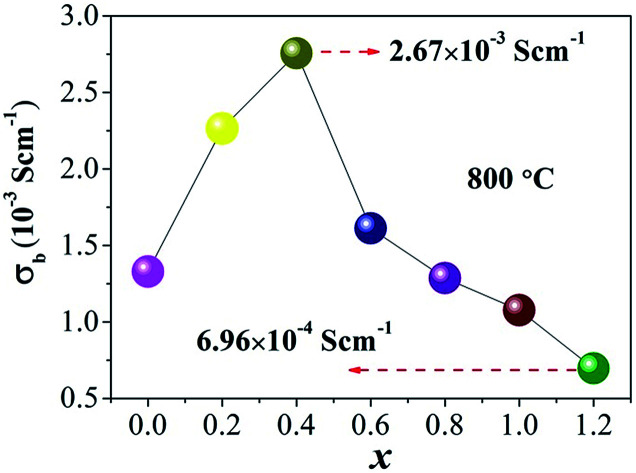

## 1. Introduction

Solid oxide fuel cells (SOFCs) have attracted great attention over the last few decades, due to the clean energy conversion technology associated with high efficiency and fuel flexibility.^1–3^ The oxide ion conducting electrolyte plays a key role in the working temperature of a SOFC.^4–6^ The yttrium-stabilized ZrO_2_ (YSZ), as the traditional and most widely used electrolyte in industry, can be applied only at a temperature higher than 750 °C.^7–9^ This high-working temperature raises issues concerning undesired reactions between the electrolyte and electrode materials, and also creates thermal stresses during thermal cycling. Thus, the development of new oxide ion conductors with considerably high conductivity for use in intermediate temperature (500–750 °C) SOFCs is an urgent and pressing need.^9–12^

**Fig. 1:**
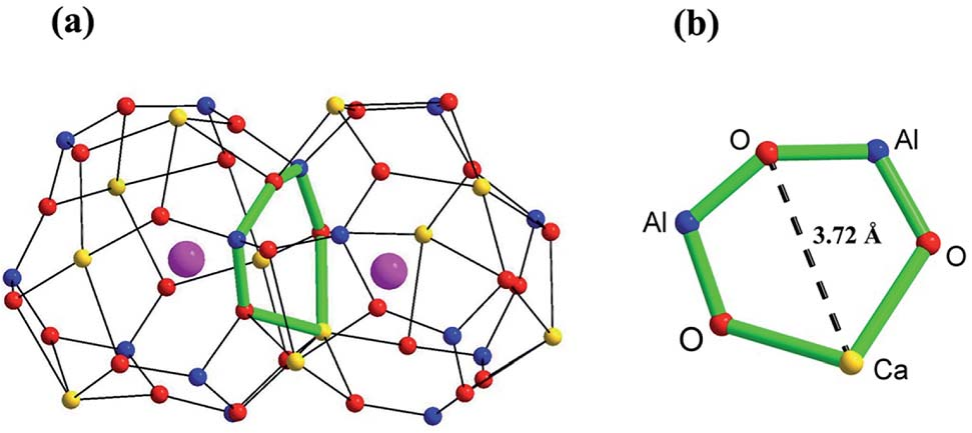
Scheme of (a) two connecting cages, and (b) the window between two cages in Ca_12_Al_14_O_33_ based on the crystallographic information reported by Boysen H. *et al.*^14^ The yellow, blue, red, and pink spheres represent Ca, Al, framework O, and free O atoms, respectively.

The oxide ion conductivity of aluminate Ca_12_Al_14_O_33_, a stable ceramic material, was first identified by M. Lacerda *et al.* in 1988.^13^ M Lacerda *et al.* reported that the oxide ion conduction (∼ 1.5 × 10^−3^ S cm^−1^) of Ca_12_Al_14_O_33_ materials was only slightly lower than that of YSZ, making Ca_12_Al_14_O_33_ a competitive candidate for SOFC electrolyte, if the oxide ion conductivity could be further improved through metal cationic doping strategy. The parent Ca_12_Al_14_O_33_ adopts a cubic structure with the *I*4̄3*d* space group and a lattice constant of ∼11.99 Å. There are two Ca_12_Al_14_O_33_ molecules in the unit cell which is composed of a positively charged framework built from 12 cages and two free O ions randomly occupying 2 different cages. Thus, it can also be represented by the chemical formula [Ca_24_Al_28_O_64_]^4+^·2O^2−^. The cages, composed of framework Ca, Al (6-coordinated Al1 and 4-coordinated Al2) and O atoms, are approximately 6 Å wide and connected to 8 other cages *via* ∼3.7 Å wide windows (Fig. 1). The free oxygen ions possess high mobility, which makes the un-doped material somewhat of an oxide-ion conductor. The oxygen diffusion in un-doped mayenite had been examined by neutron diffraction,^14,15^ DFT and MD calculations^16,17^ and oxygen isotope exchange experiments.^18,19^ The high-temperature neutron diffraction study on the un-doped Ca_12_Al_14_O_33_ suggested that the ‘free’ oxygen ions are most likely transported *via* a jump-like process involving exchange of the ‘free’ oxygen with framework oxygen.^14^ This was consistent with the theoretical prediction reported by Sushko *et al.*^17^ Irvine and West *et al.* made the first attempts at improving the high-temperature conductivity by zinc doping, as well as with zinc and phosphorus co-doping on Al atom sites.^20^ They found that for the zinc-only series compositions, replacement of aluminium by zinc would cause a decrease in conductivity (∼6.5 × 10^−4^ S cm^−1^ at 700 °C) and an increasing in activation energy. Similar electrical behaviors were also found for the zinc and phosphorus co-doped compositions. Later, G. Ebbinghaus *et al.* studied iron-doped single crystal mayenite in which they observed slight decreases in conductivity (∼1.26 × 10^−3^ S cm^−1^ at 700 °C, while the conductivity of the un-doped single crystal Ca_12_Al_14_O_33_ is ∼1.78 × 10^−3^ S cm^−1^) for the doped samples.^21^ Other works have included copper,^22,23^ nickel,^24^ gallium,^25^ manganese,^26^ and iridium^27^ incorporation into mayenite, but did not examine the effect of these dopants on the conductivity. Some other cations doped materials, such as Bi^3+^ and Ln^3+^(Tb^3+^/Sm^3+^/Er^3+^/Nd^3+^/Yb^3+^/Ho^3+^/Pr^3+^) substituting for Ca ions, were also reported and focused mainly on the luminescent properties,^28,29^ without oxide ion conductivities being investigated.

Besides metal cations doping, substituting the free oxygen ions with other anions such as F^−^, OH^−^, O_2_^−^, H^−^, O^−^, and so on,^30–36^ had also been reported with some interesting properties. In addition, electrons can occupy the empty space inside each cage in a similar manner, forming electride materials. However, although these anions or just electrons substituted Ca_12_Al_14_O_33_-based materials may have much higher conductivity, such as the H^−^ introduced and photo-activated material had an electrical conductivity as high as 0.3 S cm^−1^ (mainly n-type electronic conduction) at room temperature, the oxide ion conduction was not improved, and therefore did not benefit its application in SOFCs as electrolyte. These anions or electrons substituted Ca_12_Al_14_O_33_-based materials are thus out of the scope of our study interesting.

As mentioned above, the gallium doped Ca_12_Al_14_O_33_ materials have been previously reported by Luis Palacios *et al.* with the structures and reduction behaviors being studied.^25^ Through the Rietveld refinements based on the combined neutron and X-ray powder diffraction data, unit cell expansions were observed for these doped materials and the Ga ions were reported to mainly occupy the 4-coordinated Al2 sites. In order to get Ga-doped Ca_12_Al_14_O_33_ electrides, they fired these doped materials under a strong reducing condition, but resulted finally in decompositions and forming a mixture of Ca_12_Al_14_O_33_, Ca_3_Al_2_O_6_, and amorphous Ga metal. In this study we focused on investigating the effects of Ga doping on the phase, defect formation energy, structure and electrical properties of Ca_12_Al_14_O_33_ by X-ray diffraction, SEM/EDS, static lattice atomistic simulation, and AC impedance spectroscopy techniques. The results show that Ga ions can be substituted for up to about ten percent of Al ions in the crystal structure, similar with that reported in Luis Palacios *et al.*’s work. However, we found that both the Rietveld refinements based on the high-resolution XRD data and the defect formation energy calculations, did not suggest a preferred occupation for Ga ions replacing the two distinguishable Al1 and Al2 sites. This is different from that reported by Luis Palacios *et al.* The bulk electrical conduction of Ca_12_Al_14−*x*_Ga_*x*_O_33+*δ*_ was increased for Ga content in the range of 0 ≤ *x* ≤ 0.4, after which a decrease in the conductivity was observed for *x* > 0.4.

## 2. Methods

The samples of Ca_12_Al_14−*x*_Ga_*x*_O_33+*δ*_ were prepared by traditional solid-state reaction method using stoichiometric amounts of CaCO_3_ (Alfa Aesar, > 99.8% purity), Al_2_O_3_ (Alfa Aesar, > 99.997% purity) and Ga_2_O_3_ (Alfa Aesar, > 99.99% purity) as the starting raw materials. First, the well-mixed and ground raw materials were fired at 1000 °C for 12 h. After regrinding, the calcined powders were then uniaxially pressed into pellets and sintered at 1300 °C for 24 hours in an air atmosphere. The densities of these prepared ceramics were estimated from the samples' weight and geometry.

The XRD data were collected on a Panalytical X'pert Pro X-ray diffractometer with Cu Kα radiation over a 2*θ* range of 5−120°. The variable temperature XRD measurements were performed over a temperature range 25–900 °C with the 2*θ* range of 10−80 °C. The Rietveld refinements of the XRD data were carried out using Topas-Academic software.^37^ The microstructure and EDS analyses were performed on a Hitachi (Tokyo, Japan) S4800 scanning electron microscope (SEM). Before the SEM/EDS measurements, all these as-made ceramic pellets were well polished, followed with thermal etched. AC impedance spectroscopy (IS) measurements were performed with a Solartron 1260 frequency response analyzer over a 10^7^ to 10^−1^ Hz frequency range. Prior to the IS measurements, electrodes were formed by coating platinum paste on opposite faces of the pellets and fired at 750 °C for 3 h to remove any organic components. Before the impedance measurements, the temperature was equilibrated at each set point for 1 hour.

The energies of Ga^3+^ ions substituting for Al^3+^ ions were investigated through atomistic-static-lattice simulation, using the General Utility Lattice Program (GULP)^38,39^ based on interatomic potential approach. In this work, the Buckingham potential function^40^ was used to model the interaction between ions with the shell model^41^ to describe the electronic polarizability for the structural modeling. The interatomic potential parameters used for the atomistic simulation are listed in Table 1.

**Table 1:** Buckingham interatomic potentials and shell model parameters used for atomistic GULP simulation

Interaction	*A* (eV)	*ρ* (Å)	*C* (eV Å^6^)	*Y* (e)	*k* (eV Å^−2^)
Ca^2+^–O^2−^	1227.7	0.3372	0.0	0	—
Al^3+^–O^2−^	1474.4	0.3006	0.0	1.458	1732.0
Ga^3+^–O^2−^	1625.72	0.3019	0.0	0	—
O^2−^–O^2−^	9547.96	0.2191	32	−2.869	42.0

## 3. Results and discussion

### 3.1. Phases and structure analysis

**Fig. 2:**
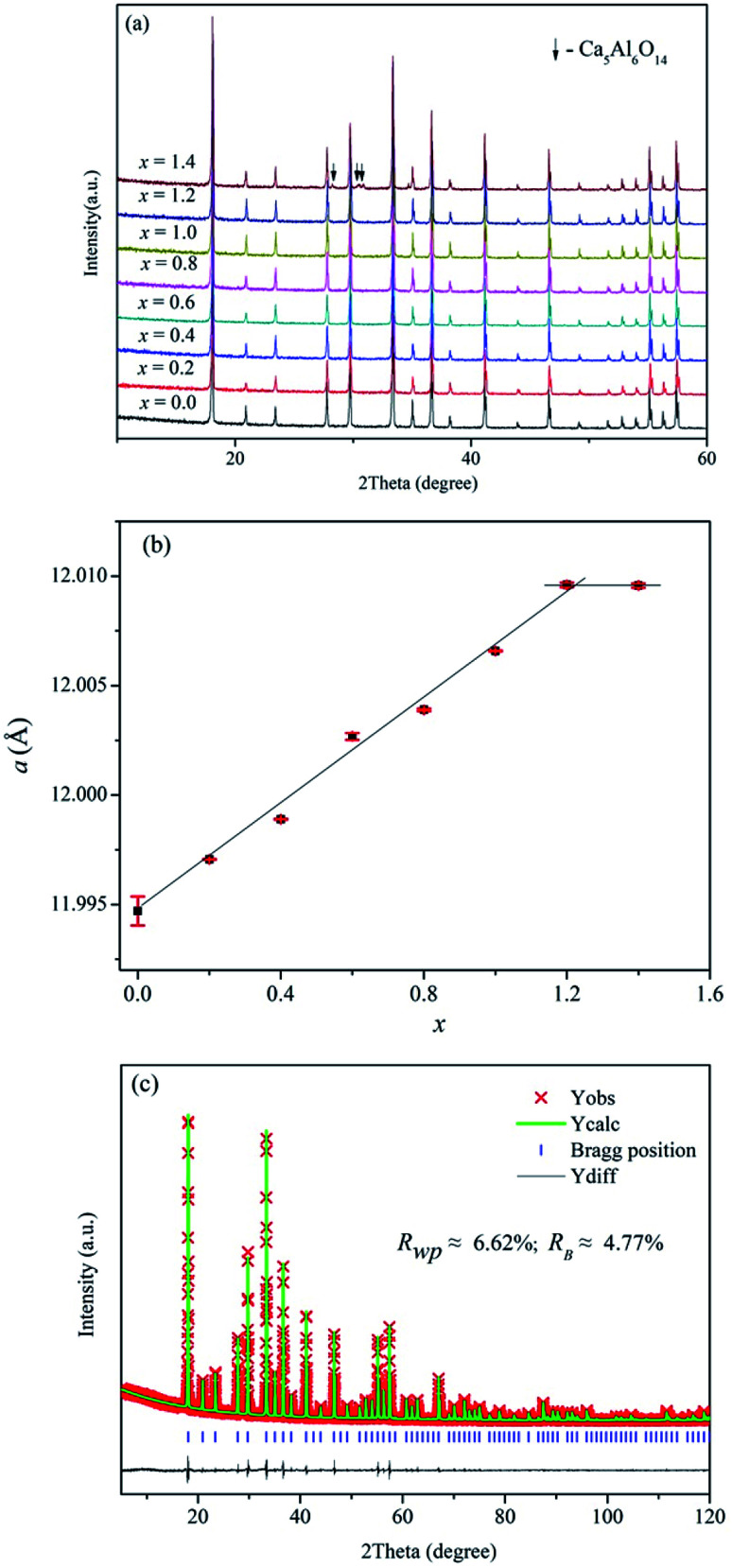
(a) XRD patterns, (b) refined cell parameters of as-made Ca_12_Al_14−*x*_Ga_*x*_O_33+*δ*_, and (c) typical Rietveld fitting plot for the composition Ca_12_Al_12.8_Ga_1.2_O_33+*δ*_.

Fig. 2a displays the XRD patterns of Ca_12_Al_14−*x*_Ga_*x*_O_33+*δ*_ (0 ≤ *x* ≤ 1.4). We can see clearly that pure phase can be obtained for compositions *x* ≤ 1.2, and all the reflection peaks match well with the mayenite structure Ca_12_Al_14_O_33_ (PDF#70-2144). For *x* = 1.4, reflections from the impurity Ca_5_Al_6_O_14_ (PDF#11-0357) appear. This solid solution limit is close to that (*x* = 1.0) reported by Luis Palacios *et al.*^25^ The refined cell parameters of different composition were plotted in Fig. 2b which shows a linear increase with the Ga content for *x* ≤ 1.2, obeying the Vegard's law; similar cell lattice expansion for these Ga-doped Ca_12_Al_14_O_33_ materials was also observed in Luis Palacios *et al.*’s work. The cell parameters of *x* = 1.4 are nearly the same as that of *x* = 1.2, indicating the solid solution limit being close to *x* = 1.2. The linear increase for *x* ≤ 1.2 can be explained by the larger effective ionic radius of Ga^3+^ ions (0.47 Å or 0.62 Å in a 4-coordinated or 6-coordinated environment, respectively) than that of Al^3+^ ions (0.39 Å or 0.53 Å in a 4-coordinated or 6-coordinated environment, respectively).^42^ Rietveld structure refinements were subsequently carried out on these doped materials. For these Rietveld refinements, a parent cubic Ca_12_Al_14_O_33_ structure (space group *I*4̄3*d*) containing two Ca sites (24d), one 3-linked and one 4-linked Al site (16c and 12a, respectively, with the numbers 3 and 4 denoting the oxygen number of a AlO_4_ tetrahedron that corner-shared with other AlO_4_ tetrahedra) and three oxygen sites (one 16c and two 48e) was used as a model. Here, a typical Rietveld refinement was described for the *x* = 1.2 composition which has the highest nominal Ga content, while refinements for other single phase compositions were provided in the ESI of this paper (Fig. S1–S6 and Tables S1–S6).† The refinement was conduct with the background, lattice parameters, peak shape parameters refined in sequence. This was followed by the positional parameters and then isotropic thermal vibration parameters being refined, which resulted in an acceptable agreements between the measured and calculated diffraction patterns. Finally, the ratios of Ga and Al on the 6-coordinated and 4-coordinated positions were varied, subject to the constraint that both sites remained fully occupied. The final fitted plot and structural parameters are given in Fig. 2c and Table 2, respectively. It can be seen that the Ga ions show essentially a random distribution among the Al1 and Al2 sites, which is different from the case reported by Luis Palacios *et al.* that the Ga ions shown a preferred occupation on the 4-coordinated position. The refined chemical formula of Ca_12_Al_12.784(2)_Ga_1.216(2)_O_33+*δ*_ agrees well with the nominal composition of Ca_12_Al_12.8_Ga_1.2_O_33_.

**Table 2:** Final refined structural parameters of nominal Ca_12_Al_12.8_Ga_1.2_O_33+*δ*_. Lattice parameters: *a* = 12.0019(1) Å, space group *I*4̄3*d*

Atom	Site	*x*	*y*	*z*	Occupancy	*B*_iso_ (Å^2^)
Ca1	24d	0.1074(1)	0	1/4	0.851(2)	0.53(2)
Ca2	24d	0.036(1)	0	1/4	0.149(2)	2.6(1)
Al1/Ga1	12a	3/8	0	1/4	0.908(2)/0.092(1)	2.23(2)
Al2/Ga2	16c	−0.0156(2)	−0.0156(2)	−0.0156(2)	0.917(1)/0.083(1)	1.32(1)
O1	16c	0.0579(3)	0.0579(3)	0.0579(3)	1	1.2(1)
O2	48e	0.1025(3)	0.1916(1)	0.2864(2)	1	1.33(4)
O3	48e	0.256(2)	0.160(1)	0.984(3)	0.0416(1)	1.15(2)

**Fig. 3:**
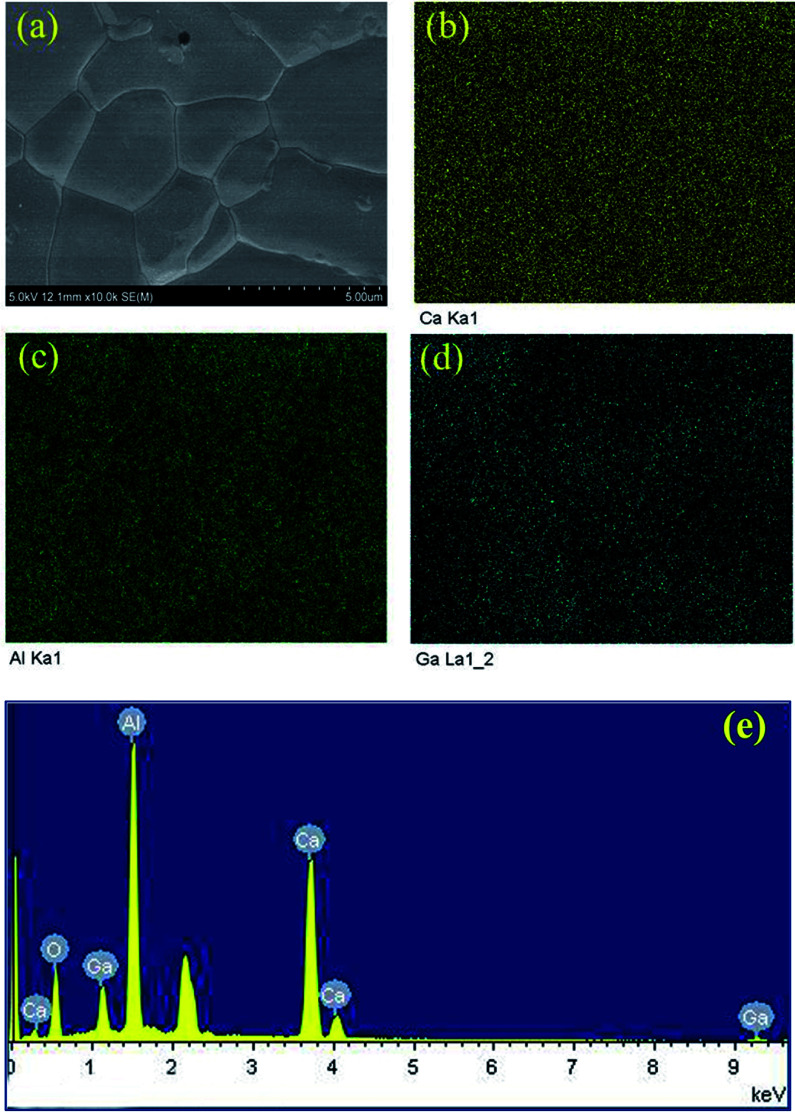
SEM micrograph of Ca_12_Al_12.8_Ga_1.2_O_33+*δ*_ (a), and EDS element distribution maps of Ca (b), Al (c) and Ga (d); picture (e) shows the element concentrations, the un-labeled peak is ascribed to the Au element that sprayed on the surface of the ceramic pellet before measurements.

Fig. 3a demonstrates the typical SEM micrograph image of the composition *x* = 1.2, confirming the dense structure of this prepared ceramic. The grain size ranges from 2 μm to 5.0 μm, with irregular morphology. Fig. 3b–d display the EDS element distribution maps of elements Ca, Al, and Ga, respectively. We can see that all these elements show homogeneous distributions, indicating the pure phase nature of the ceramic. This is consistent with the results from XRD data analysis. Fig. 3e shows the element concentrations and reveals a relative ratios for Ca : Al : Ga to be 12 : 13.91 : 1.4, close to the nominal ratios. The SEM and EDS results for other compositions are provided in Fig. S7–S12.†

### 3.2. Static lattice simulation

As stated previously, no more than 10 percent of the Al ions can be replaced by Ga ions. This narrow solid solution usually corresponds with the relatively high defect formation energy for substitution. To confirm this, static lattice simulation technology was used to calculate defect formation energies based on the appropriate combination of dopant and vacancy defect energies and lattice energies of the binary oxides, and can be determined using the following defect equation:1
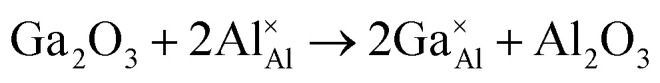
*i.e.*,2
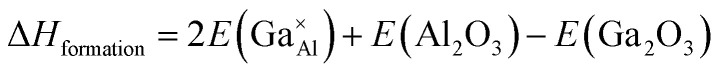
where *E*(*X*) is the calculated total lattice energy or point defect energy of the species of interest after geometry optimization.

The starting point of the study was to reproduce the experimentally observed crystal structures of Ca_12_Al_14_O_33_, Ga_2_O_3_, and Al_2_O_3_. Using the interatomic potentials presented in Table 1 for simulation, the differences between the calculated and experimental unit cell edges and volumes for all three oxides were found to be less than 4%, as demonstrated in Table 3, validating the rationality of these interatomic potentials used for simulations.

**Table 3:** Calculated and experimental structural parameters of Ca_12_Al_14_O_33_ (space group *I*4̄3*d*), Ga_2_O_3_ (space group *R*3̄*cH*) and Al_2_O_3_ (space group *R*3̄*cH*)

Oxides	Parameters	Experimental	Calculated	Difference	Percent (%)
Ca_12_Al_14_O_33_	*a*/*b*/*c* (Å)	12.04	12.092	0.052	0.43
	*α*/*β*/*γ* (degree)	90	90	0	0
	Volume (Å^3^)	1745.34	1768.15	22.81	1.31
Ga_2_O_3_	*a*/*b* (Å)	4.9825	4.9901	0.0076	0.15
	*c* (Å)	13.4330	13.1774	−0.2556	−1.9
	*α*/*β* (degree)	90	90	0	0
	*γ* (degree)	120	120	0	0
	Volume (Å^3^)	288.8007	284.1752	−4.6255	−1.6
Al_2_O_3_	*a*/*b* (Å)	4.7540	4.8555	0.1015	2.13
	*c* (Å)	12.9900	12.8821	−0.1079	−0.83
	*α*/*β* (degree)	90	90	0	0
	*γ* (degree)	120	120	0	0
	Volume (Å^3^)	254.2483	263.0148	8.7664	3.45

Next, point defect energies for Ga ions replacing both Al1 and Al2 site ions were calculated. Combining the total lattice energies of the interested binary oxides Ga_2_O_3_ and Al_2_O_3_, the defect formation energies for Ga ions replacing Al ions can be deduced from eqn (2). These values are summarized in Table 4. It can be clearly seen that the defect formation energies for Ga ions replacing both the Al1 site and Al2 site ions are higher than 3.0 eV, consistent with the relatively narrow solid solution. In addition, the almost same defect formation energies on Al1 and Al2 sites agrees well with the randomly occupying and comparable probability of Ga atoms on the Al1 and Al2 sites, as derived from the Rietveld refinements based on the XRD data.

**Table 4:** Total lattice energies for Ga_2_O_3_ and Al_2_O_3_, point defect 
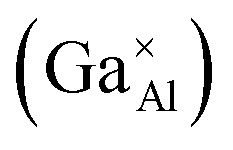
 energies in the Ca_12_Al_14_O_33_ crystal structure, and the final defect formation energies

Point defects	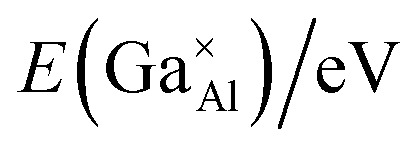	*E* (Al_2_O_3_)/eV	*E* (Ga_2_O_3_)/eV	Δ*H*_formation_/eV
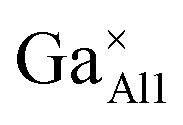	2.1202	−52.9240	−51.7161	3.03
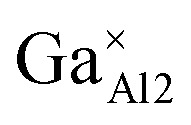	2.1257			3.04

### 3.3. Electrical properties

**Fig. 4:**
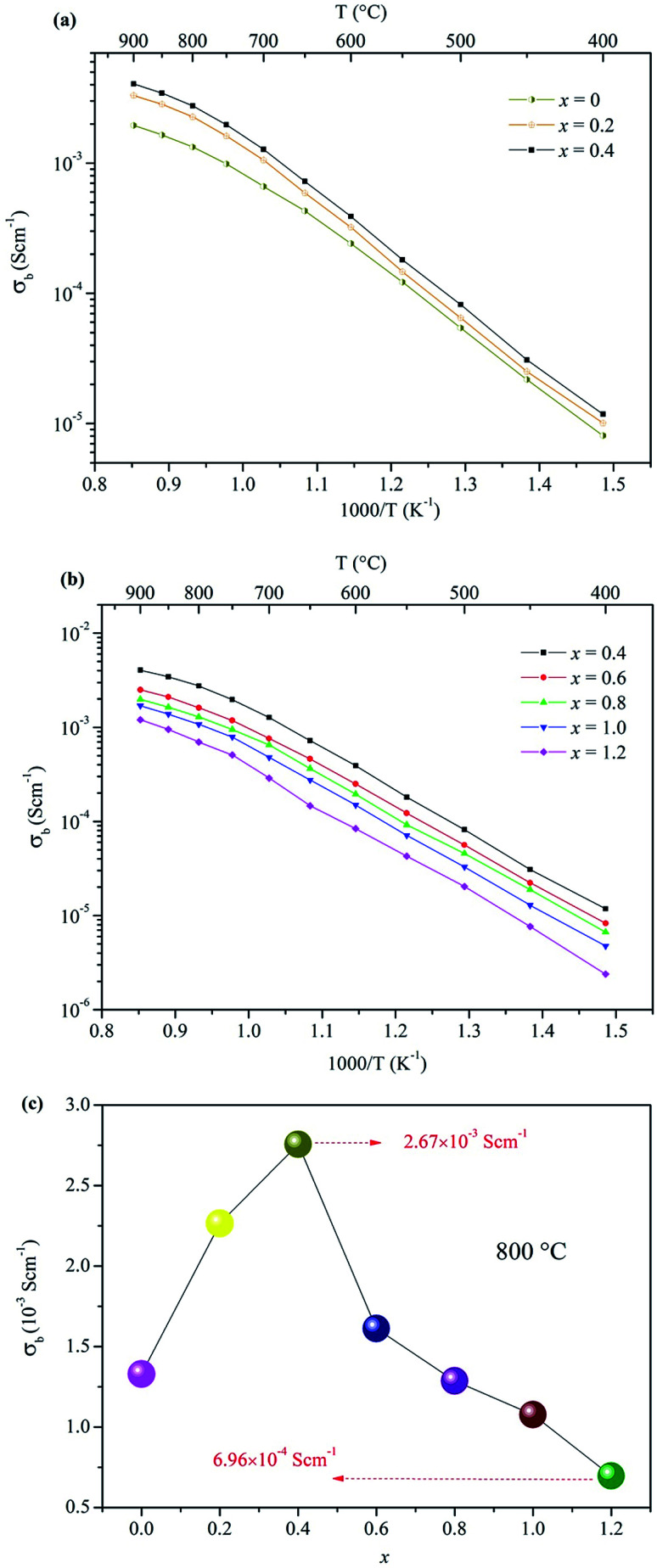
Arrhenius plots of the bulk conductivities for Ca_12_Al_14−*x*_Ga_*x*_O_33+*δ*_: (a) 0 ≤ *x* ≤ 0.4, (b) 0.4 ≤ *x* ≤ 1.2; (c) the bulk conductivities as a function of *x* at 800 °C.

The electrical properties of the Ca_12_Al_14−*x*_Ga_*x*_O_33+*δ*_ ceramic samples were investigated by AC impedance spectroscopy. Compared to four electrode technique that commonly used in the direct current (DC) conductivity measurements, the alternating current (AC) impedance spectroscopy measurements have the advantage to separate the resistance related to grains (bulk) and grain boundaries due to their different relaxation times, leading to separate semicircles in the complex impedance spectrum. Therefore, the bulk conductivity (*σ*_b_), grain boundary conductivity (*σ*_b_), and the total conductivity (*σ*_t_) can be well calculated from the bulk resistance (*R*_b_), grain boundary resistance (*R*_g_), and total resistance (*R*_t_ = *R*_b_ + *R*_g_). The densities of these prepared ceramics, estimated from the samples' weight and geometry, were all higher than 92% of the theoretical prediction. The AC impedance measurements were performed within a temperature range of 400−900 °C. The results showed that the conductivity of Ca_12_Al_14−*x*_Ga_*x*_O_33+*δ*_ ceramic samples did not vary linearly with the doped Ga content. In fact, the conductivity increased within the range 0 ≤ *x* ≤ 0.4, as shown in Fig. 4a, and then began to decrease when *x* ≥ 0.6. For *x* ≥ 0.6 the values are plotted separately in Fig. 4b for clarity. Thus, the highest conductivity was observed for the composition *x* = 0.4, with a value of ∼ 2.76 × 10^−3^ S cm^−1^ at 800 °C. For the composition *x* = 0.6, although the conductivity was lower than that of *x* = 0.4 but still higher than that of the parent material Ca_12_Al_14_O_33_. For *x* = 0.8, the measured conductivity was almost the same as that of the un-doped material. Conductivity lower than the un-doped Ca_12_Al_14_O_33_ was observed for samples with *x* = 1.0, and the lowest measured conductivity of ∼6.96 × 10^−4^ S cm^−1^ occurred for the *x* = 1.2 sample. This value, measured at 800 °C, is about four times lower than the sample with composition *x* = 0.4. A more direct visual comparison of the conductivities of the Ca_12_Al_14−*x*_Ga_*x*_O_33+*δ*_ ceramic samples is seen in Fig. 4c where the conductivity values are plotted as a function of *x* at a temperature of 800 °C. In Fig. 4c the trend of conductivities with *x* values can be clearly seen.

For all these Arrhenius plots displayed in Fig. 4a and b, one can see that there are different slops for each plot at low (≤700 °C) and high (>700 °C) temperature, respectively. The change in slop at different temperature range, however, is not caused by a phase transition, which can be validated by the *in situ* VT-XRD measurements (Fig. S13†) performed on the sample *x* = 0.4. The VT-XRD patterns show a single mayenite phase over the measured temperature range of 25–900 °C. Therefore, the slope change may originate from the variation of local defect structure that surrounding the charge carriers, in different temperature range.

## 4. Conclusion

In this work, a series of Ga-doped Ca_12_Al_14−*x*_Ga_*x*_O_33+*δ*_ (0 ≤ *x* ≤ 1.4) materials were prepared by a conventional solid state method. Pure mayenite phases were obtained for 0 ≤ *x* ≤ 1.2, as was confirmed by Rietveld refinements based on the XRD data, and also by combined SEM and EDS element distribution map and element concentrations analyses. Static lattice atomistic simulation was used to calculate the defect formation energies of Ga ions replacing Al ions. The simulation values of 3.03 eV and 3.04 eV for the defects on Al1 and Al2 sites, respectively, were consistent with the non-preferred occupation of Ga on Al1 or Al2 sites, as derived from Rietveld refinements. The bulk conductivities increased with Ga content for 0 ≤ *x* ≤ 0.4. In summary, we believe that this paper presents the first example of successful improvement of the bulk oxide ion conductivity of Ca_12_Al_14_O_33_ through Ga-doping.

## Conflicts of interest

There are no conflicts of interest to declare.

## Supplementary Material

RA-009-C8RA08254E-s001
