# Supplementary material for: Ga-doped Ca12Al14O33 mayenite oxide ion conductors: synthesis, defects, and electrical properties
Source: RSC Adv. 2019 Jan 30;9(7):3809–15. doi: 10.1039/c8ra08254e (PMC9060535; doi:10.1039/c8ra08254e)
Supplement: RA-009-C8RA08254E-s001 [file RA-009-C8RA08254E-s001.pdf]

Electronic Supplementary Material (ESI) for RSC Advances.
This journal is © The Royal Society of Chemistry 2019

Supplementary information for

Ga-doped $Ca_{12}Al_{14}O_{33}$ mayenite oxide ion conductors: synthesis, defects, and electrical properties

Huaibo Yi[1], Yun Lv[1], Yanhui Wang[1], Xue Fang[1], Victoria Mattick[2], Jungu Xu[1, *]

1. *MOE Key Laboratory of New Processing Technology for Nonferrous Metals and Materials, Guangxi Universities Key Laboratory of Non-ferrous Metal Oxide Electronic Functional Materials and Devices, College of Materials Science and Engineering, Guilin University of Technology, Guilin 541004, P. R. China*

2. *Department of Chemical Engineering, University of South Carolina, Columbia, SC 29201, USA*

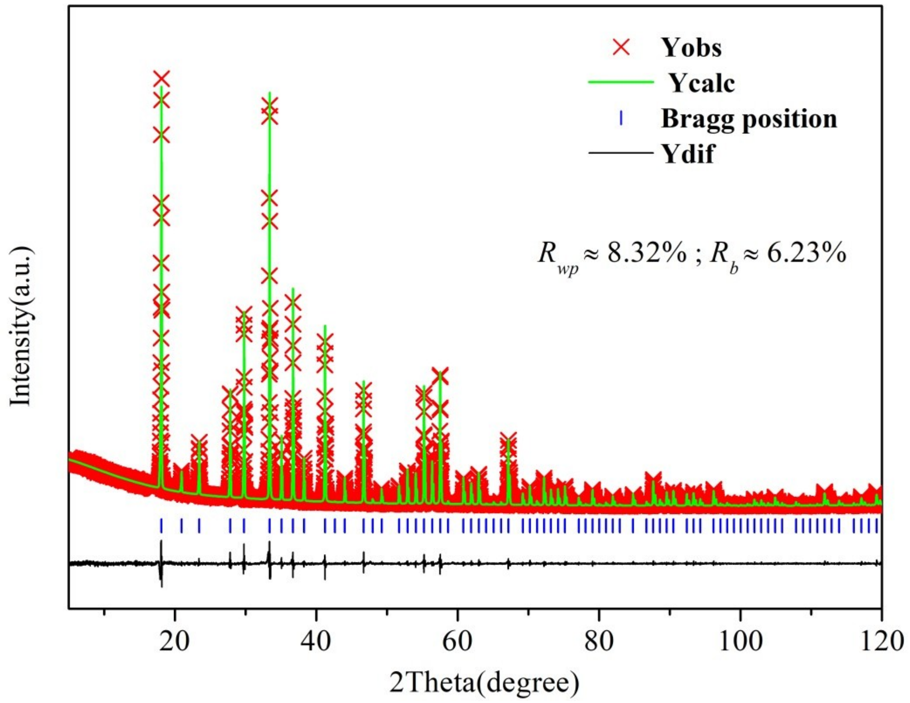

Figure S1. Rietveld fitting plot for the parent $Ca_{12}Al_{14}O_{33}$.

Table S1. Final refined structural parameters of parent $Ca_{12}Al_{14}O_{33}$. Lattice parameters: a =11.9947(6) Å, space group $I\bar{4}3d$.

Atom	Site	*x*	*y*	*z*	Occupies	B_{iso}(Å^2)
Ca1	24d	0.1107(5)	0	1/4	0.862(3)	1.45(1)
Ca2	24d	0.041(1)	0	1/4	0.138(3)	3.0(2)
Al1	12a	3/8	0	1/4	1	0.37(8)
Al2	16c	-0.01811(9)	-0.01811(9)	-0.01811(9)	1	0.74(6)
O1	16c	0.0609(2)	0.0609(2)	0.0609(2)	1	1.6(1)
O2	48e	0.1033(2)	0.1949(2)	0.2874(2)	1	3.0(1)
O3	48e	0.267(9)	1.143(5)	1.00(1)	0.0416(1)	0.8(1)

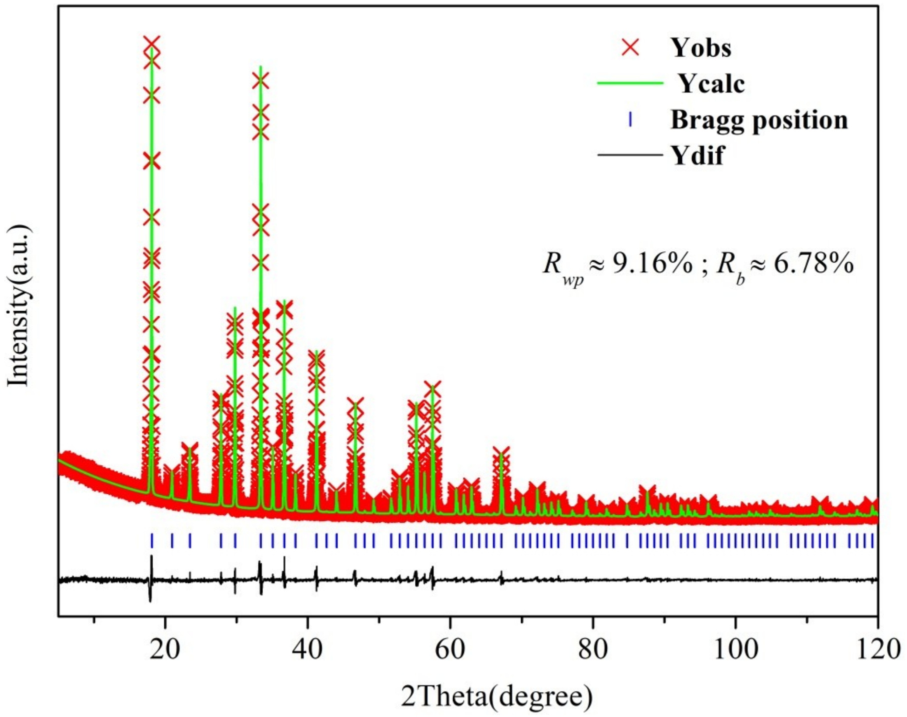

Figure S2. Rietveld fitting plot for the composition $Ca_{12}Al_{13.8}Ga_{0.2}O_{33+\delta}$.

Table S2. Final refined structural parameters of the composition $Ca_{12}Al_{13.8}Ga_{0.2}O_{33+\delta}$. Lattice parameters: a =11.9970(1) Å, space group $I\bar{4}3d$.

Atom	Site	*x*	*y*	*z*	Occupies	B_{iso}(Å^2)
Ca1	24d	0.1063(1)	0	1/4	0.851(3)	0.66(5)
Ca2	24d	0.039(1)	0	1/4	0.149(3)	2.3(3)
Al1/Ga1	12a	3/8	0	1/4	0.982(1)/0.018(1)	2.2(1)
Al2/Ga2	16c	-0.0155(1)	-0.0155(1)	-0.0155(1)	0.986(3)/0.014(3)	2.47(8)
O1	16c	0.0613(3)	0.0613(3)	0.0613(3)	1	2.5(2)
O2	48e	0.1029(2)	0.1918(2)	0.2856(2)	1	1.34(9)
O3	48e	0.26(1)	0.164(5)	1.009(6)	0.0416(1)	1.03(2)

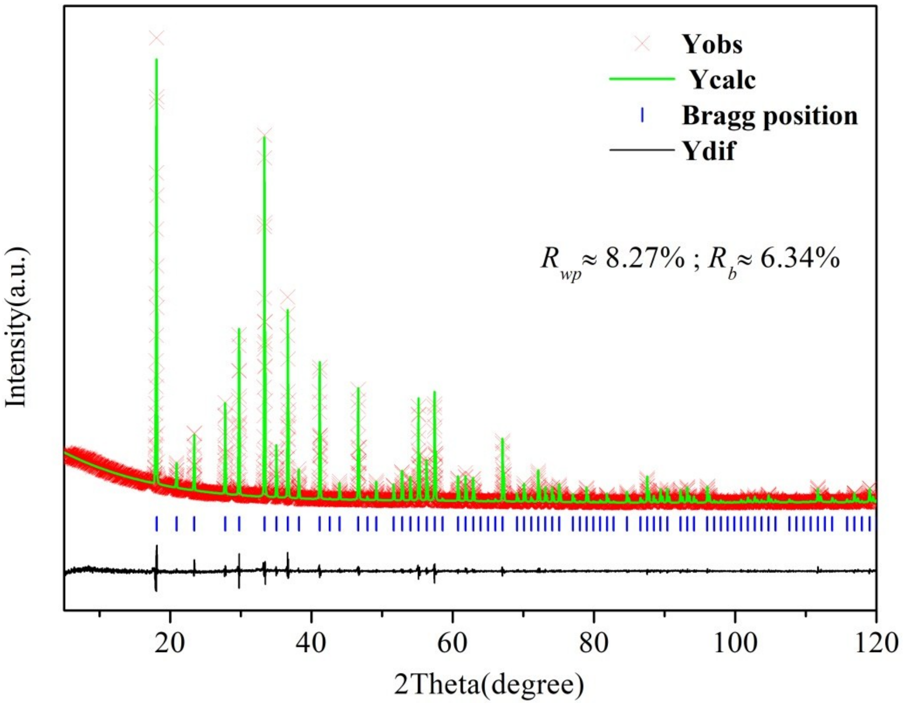

Figure S3. Rietveld fitting plot for the composition $Ca_{12}Al_{13.6}Ga_{0.4}O_{33+\delta}$.

Table S3. Final refined structural parameters of the composition $Ca_{12}Al_{13.6}Ga_{0.4}O_{33+\delta}$. Lattice parameters: a =11.9989(3) Å, space group $I\bar{4}3d$.

Atom	Site	*x*	*y*	*z*	Occupies	B_{iso}(Å^2)
Ca1	24d	0.1062(4)	0	1/4	0.849(4)	0.66(7)
Ca2	24d	0.040(6)	0	1/4	0.151(4)	2.3(1)
Al1/Ga1	12a	3/8	0	1/4	0.966(1)/0.034(1)	2.2(1)
Al2/Ga2	16c	-0.0162(1)	-0.0162(1)	-0.0162(1)	0.974(2)/0.026(2)	2.4(1)
O1	16c	0.0578(3)	0.0578(3)	0.0578(3)	1	1.2(1)
O2	48e	0.1033(3)	0.1924(1)	0.2871(4)	1	0.56(3)
O3	48e	0.255(3)	0.161(8)	1.03(2)	0.0416(1)	2.53(2)

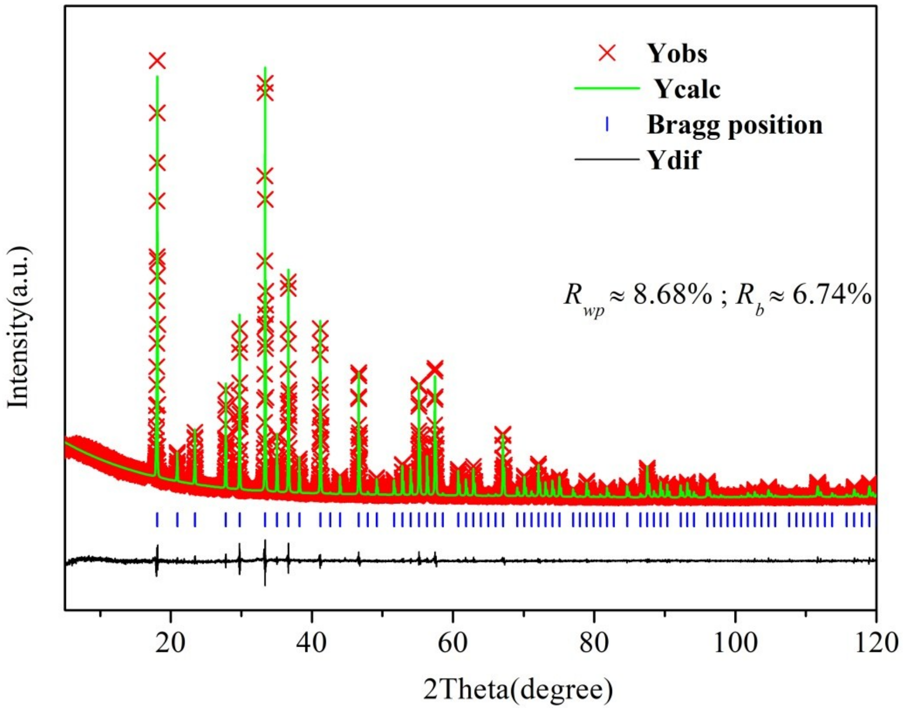

Figure S4. Rietveld fitting plot for the composition $Ca_{12}Al_{13.4}Ga_{0.6}O_{33+\delta}$.

Table S4. Final refined structural parameters of the composition $Ca_{12}Al_{13.4}Ga_{0.6}O_{33+\delta}$. Lattice parameters: a =12.0026(1) Å, space group $I\bar{4}3d$.

Atom	Site	*x*	*y*	*z*	Occupies	B_{iso}(Å^2)
Ca1	24d	0.1061(3)	0	1/4	0.847(1)	0.73(3)
Ca2	24d	0.044(8)	0	1/4	0.153(1)	2.3(2)
Al1/Ga1	12a	3/8	0	1/4	0.957(1)/0.043(1)	1.39(5)
Al2/Ga2	16c	-0.0164(1)	-0.0164(1)	-0.0164(1)	0.954(3)/0.046(3)	2.69(7)
O1	16c	0.0600(3)	0.0600(3)	0.0600(3)	1	0.9(1)
O2	48e	0.1014(2)	0.1882(3)	0.2847(2)	1	2.36(8)
O3	48e	0.2(1)	1.095(3)	0.99(7)	0.0416(1)	1.65(5)

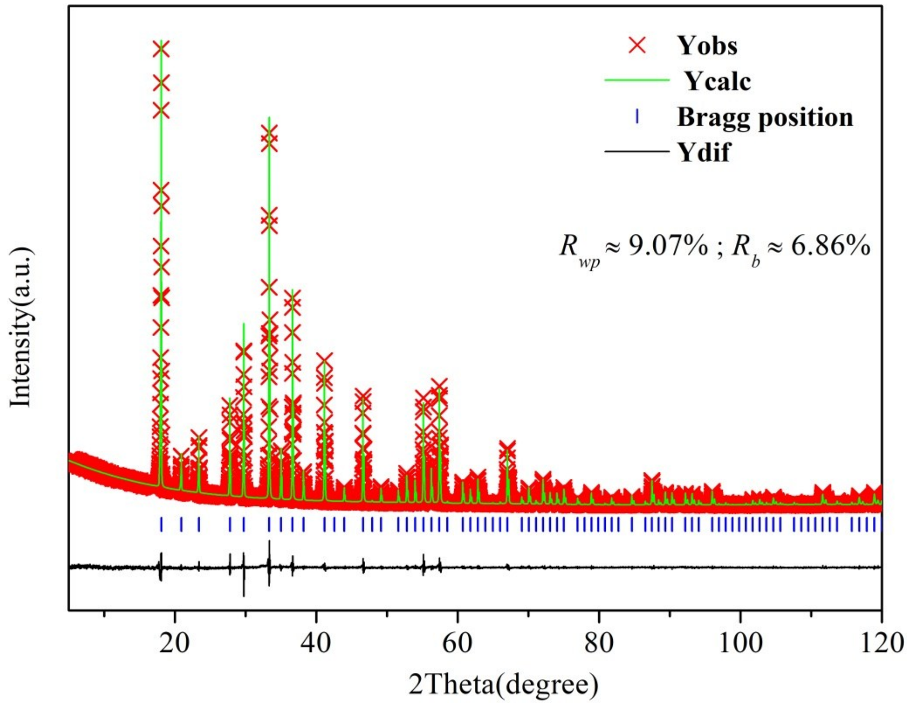

Figure S5. Rietveld fitting plot for the composition $Ca_{12}Al_{13.2}Ga_{0.8}O_{33+\delta}$.

Table S5. Final refined structural parameters of the composition $Ca_{12}Al_{13.2}Ga_{0.8}O_{33+\delta}$. Lattice parameters: a =12.0039(7) Å, space group $I\bar{4}3d$.

Atom	Site	*x*	*y*	*z*	Occupies	B_{iso}(Å^2)
Ca1	24d	0.1081(6)	0	1/4	0.849(2)	0.34(5)
Ca2	24d	0.037(1)	0	1/4	0.151(2)	2.2(1)
Al1/Ga1	12a	3/8	0	1/4	0.945(1)/0.055(1)	1.4(1)
Al2/Ga2	16c	-0.0159(1)	-0.0159(1)	-0.0159(1)	0.944(1)/0.056(1)	2.3(1)
O1	16c	0.0595(3)	0.0595(3)	0.0595(3)	1	1.4(1)
O2	48e	0.1023(2)	0.1873(2)	0.2867(1)	1	2.89(7)
O3	48e	0.25(1)	1.125(4)	1.023(7)	0.0416(1)	1.21(1)

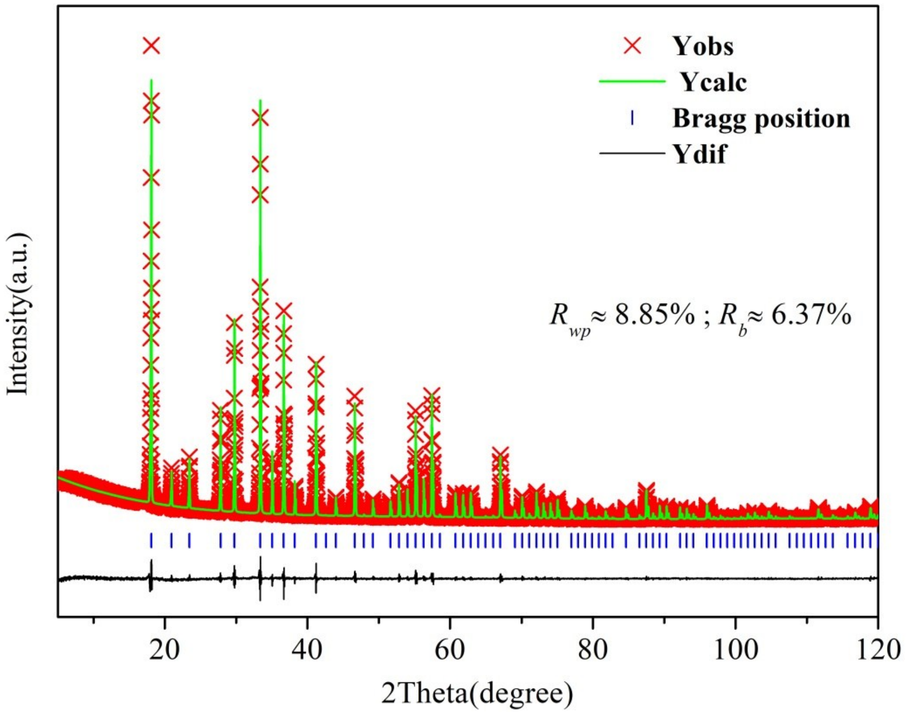

Figure S6. Rietveld fitting plot for the composition $Ca_{12}Al_{13.0}Ga_{1.0}O_{33+\delta}$.

Table S6. Final refined structural parameters of the composition $Ca_{12}Al_{13.0}Ga_{1.0}O_{33+\delta}$. Lattice parameters: a =12.0066(1) Å, space group $I\bar{4}3d$.

Atom	Site	*x*	*y*	*z*	Occupies	B_{iso}(Å^2)
Ca1	24d	0.1065(4)	0	1/4	0.837(1)	0.50(6)
Ca2	24d	0.034(1)	0	1/4	0.163(1)	0.92(7)
Al1/Ga1	12a	3/8	0	1/4	0.928(2)/0.072(2)	1.63(1)
Al2/Ga2	16c	-0.0167(1)	-0.0167(1)	-0.0167(1)	0.932(1)/0.068(1)	2.42(1)
O1	16c	0.0577(3)	0.0577(3)	0.0577(3)	1	2.6(2)
O2	48e	0.1027(2)	0.1888(2)	0.2859(2)	1	1.31(1)
O3	48e	0.245(9)	1.135(4)	0.99(1)	0.0416(1)	2.13(1)

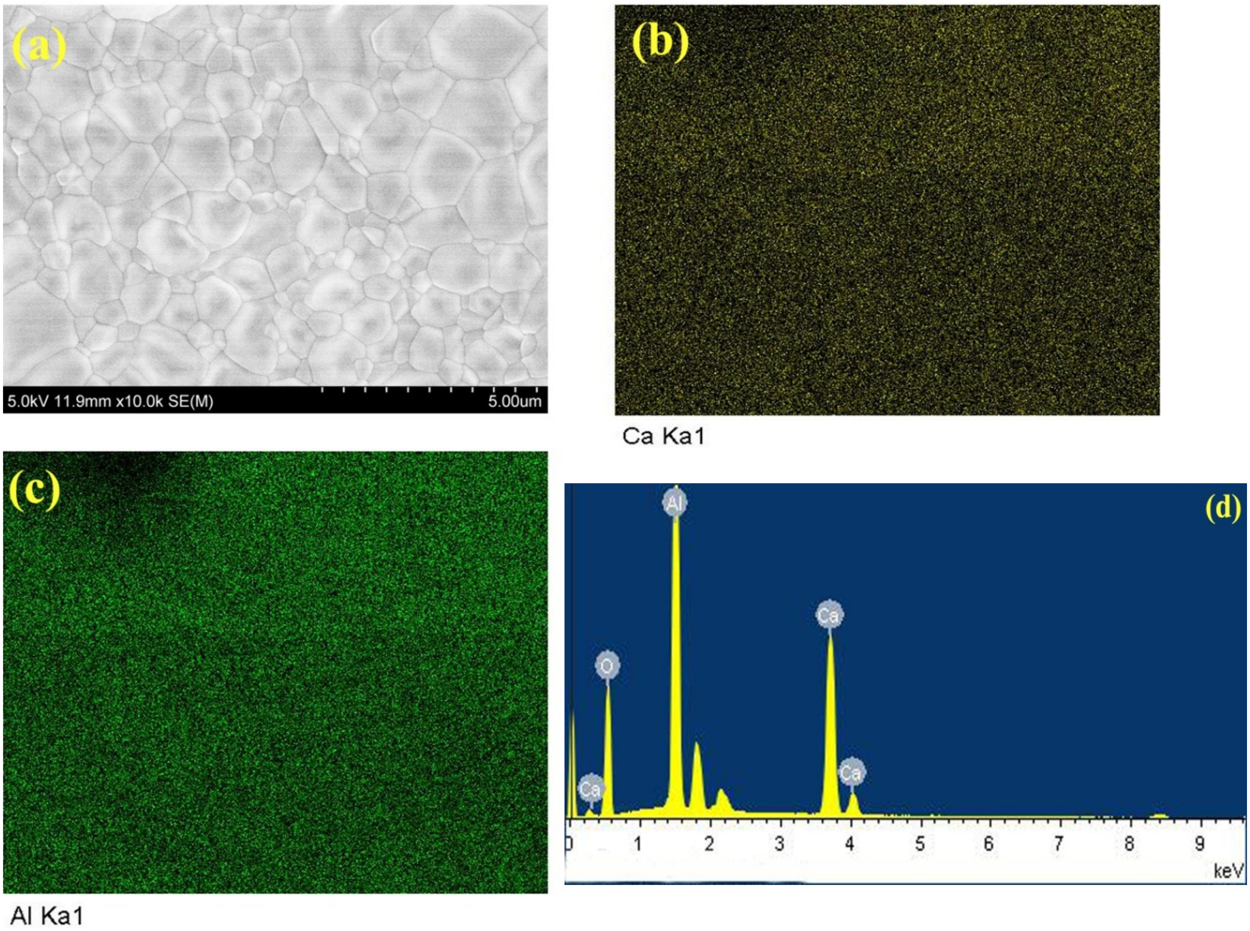

Figure S7. SEM micrograph of parent $Ca_{12}Al_{14}O_{33}$ (a), and EDS element distribution maps of Ca (b), Al (c); picture (d) shows the element concentrations, the un-labeled peak is ascribed to the Au element that sprayed on the surface of the ceramic pellet before measurements. The relative element ratio for Ca : Al was revealed to be 12 : 14.3.

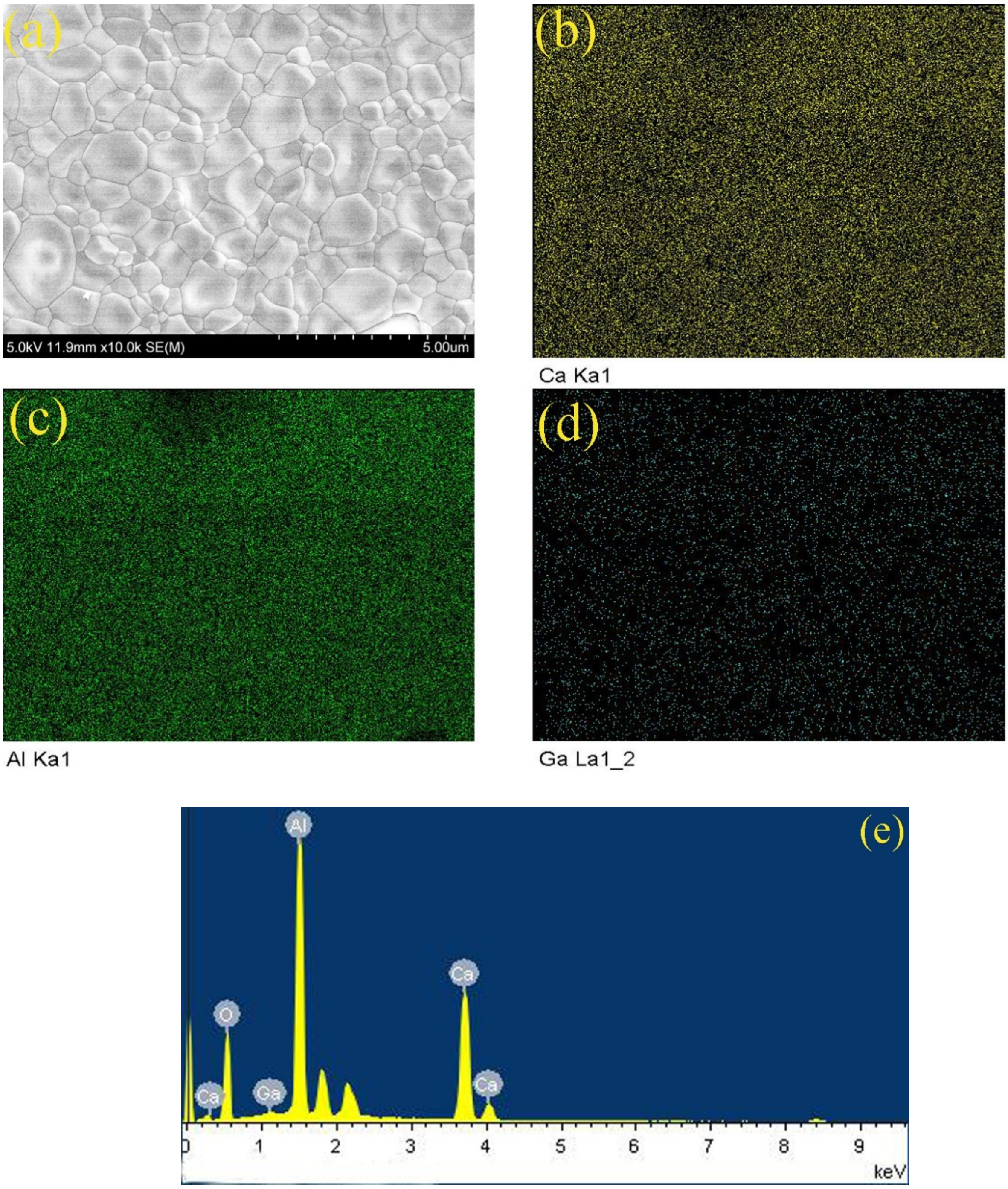

Figure S8. SEM micrograph of the composition $Ca_{12}Al_{13.8}Ga_{0.2}O_{33}$ (a), and EDS element distribution maps of Ca (b), Al (c), and Ga (d) ; picture (e) shows the element concentrations, the un-labeled peak is ascribed to the Au element that sprayed on the surface of the ceramic pellet before measurements. The relative element ratios for Ca : Al : Ga were revealed to be 12 : 13.74 : 0.23 .

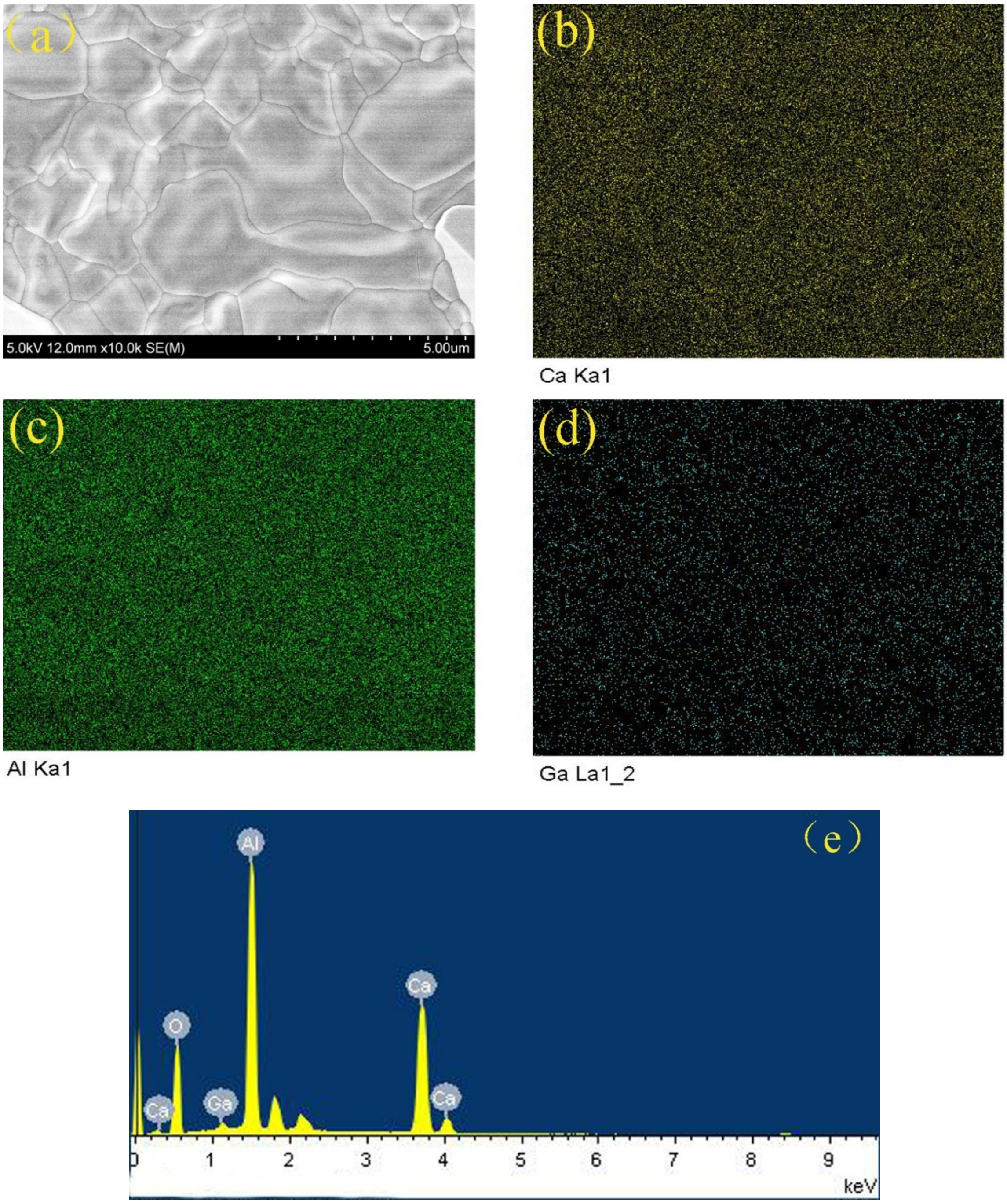

Figure S9. SEM micrograph of the composition $Ca_{12}Al_{13.6}Ga_{0.4}O_{33}$ (a), and EDS element distribution maps of Ca (b), Al (c), and Ga (d) ; picture (e) shows the element concentrations, the un-labeled peak is ascribed to the Au element that sprayed on the surface of the ceramic pellet before measurements. The relative element ratios for Ca : Al : Ga were revealed to be 12 : 13.65 : 0.36

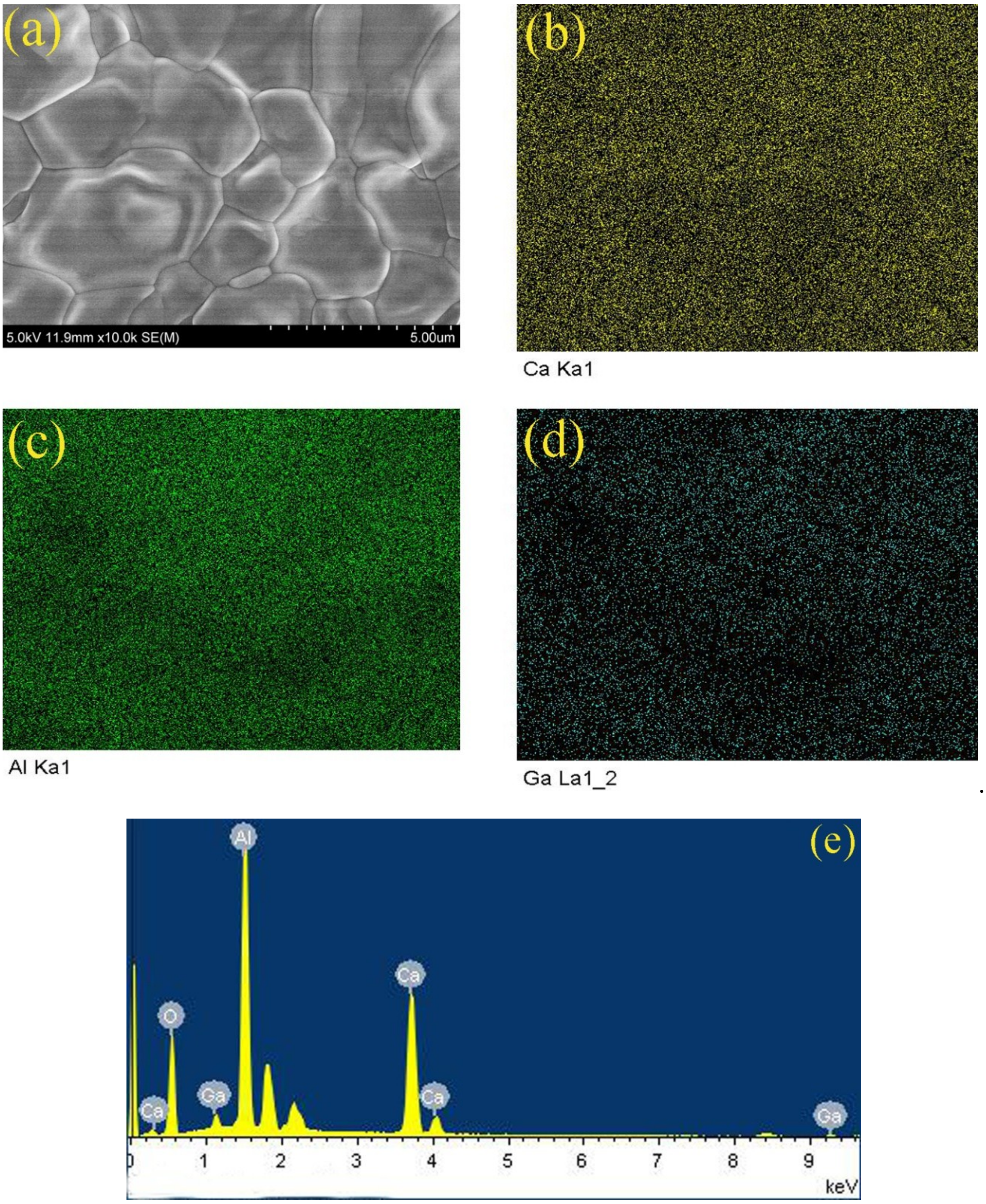

Figure S10. SEM micrograph of the composition $Ca_{12}Al_{13.4}Ga_{0.6}O_{33}$ (a), and EDS element distribution maps of Ca (b), Al (c), and Ga (d) ; picture (e) shows the element concentrations, the un-labeled peak is ascribed to the Au element that sprayed on the surface of the ceramic pellet before measurements. The relative element ratios for Ca : Al : Ga were revealed to be 12 : 13.37 : 0.62

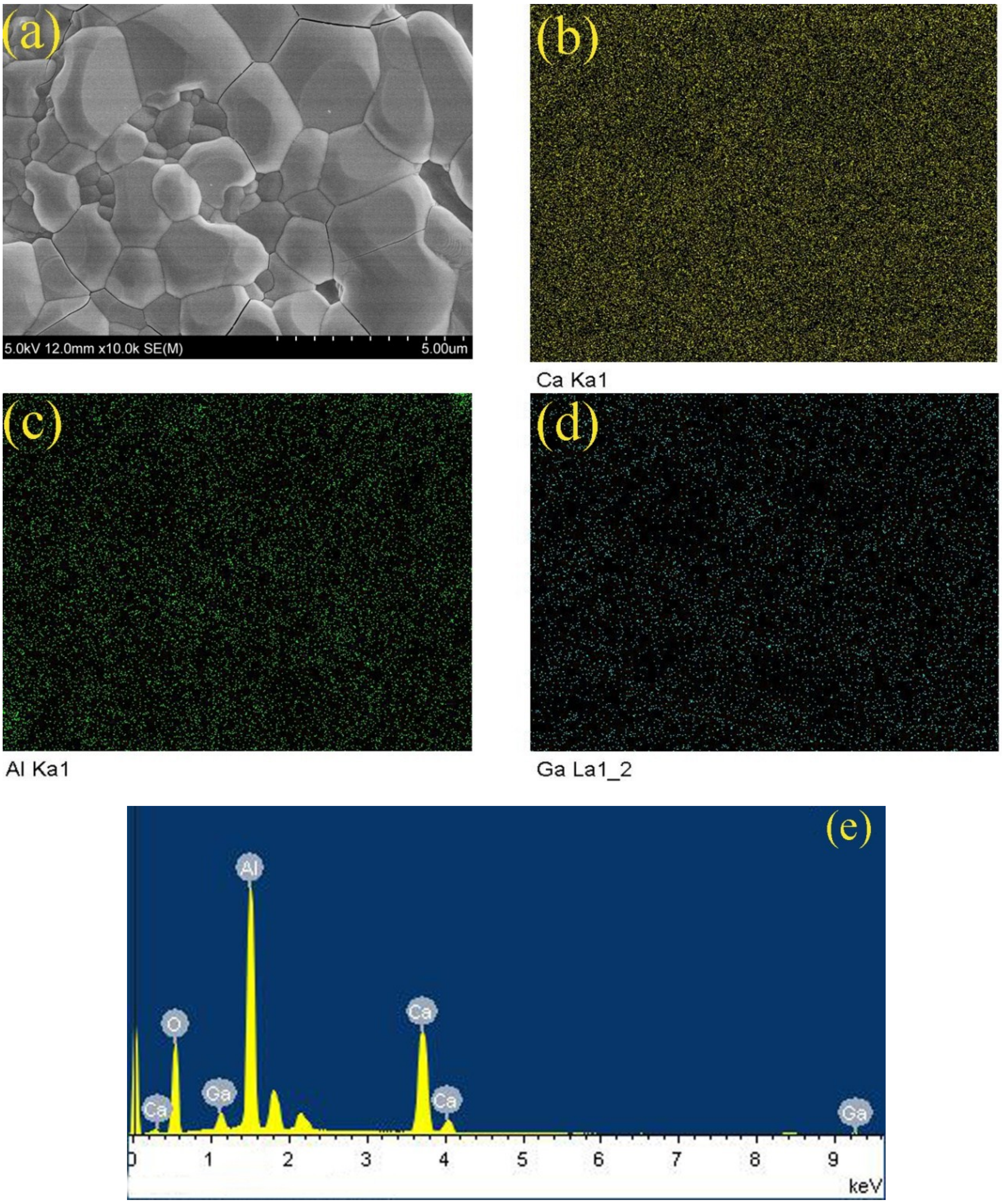

Figure S11. SEM micrograph of the composition $Ca_{12}Al_{13.2}Ga_{0.8}O_{33}$ (a), and EDS element distribution maps of Ca (b), Al (c), and Ga (d) ; picture (e) shows the element concentrations, the un-labeled peak is ascribed to the Au element that sprayed on the surface of the ceramic pellet before measurements. The relative element ratios for Ca : Al : Ga were revealed to be 12 : 13.14 : 0.81

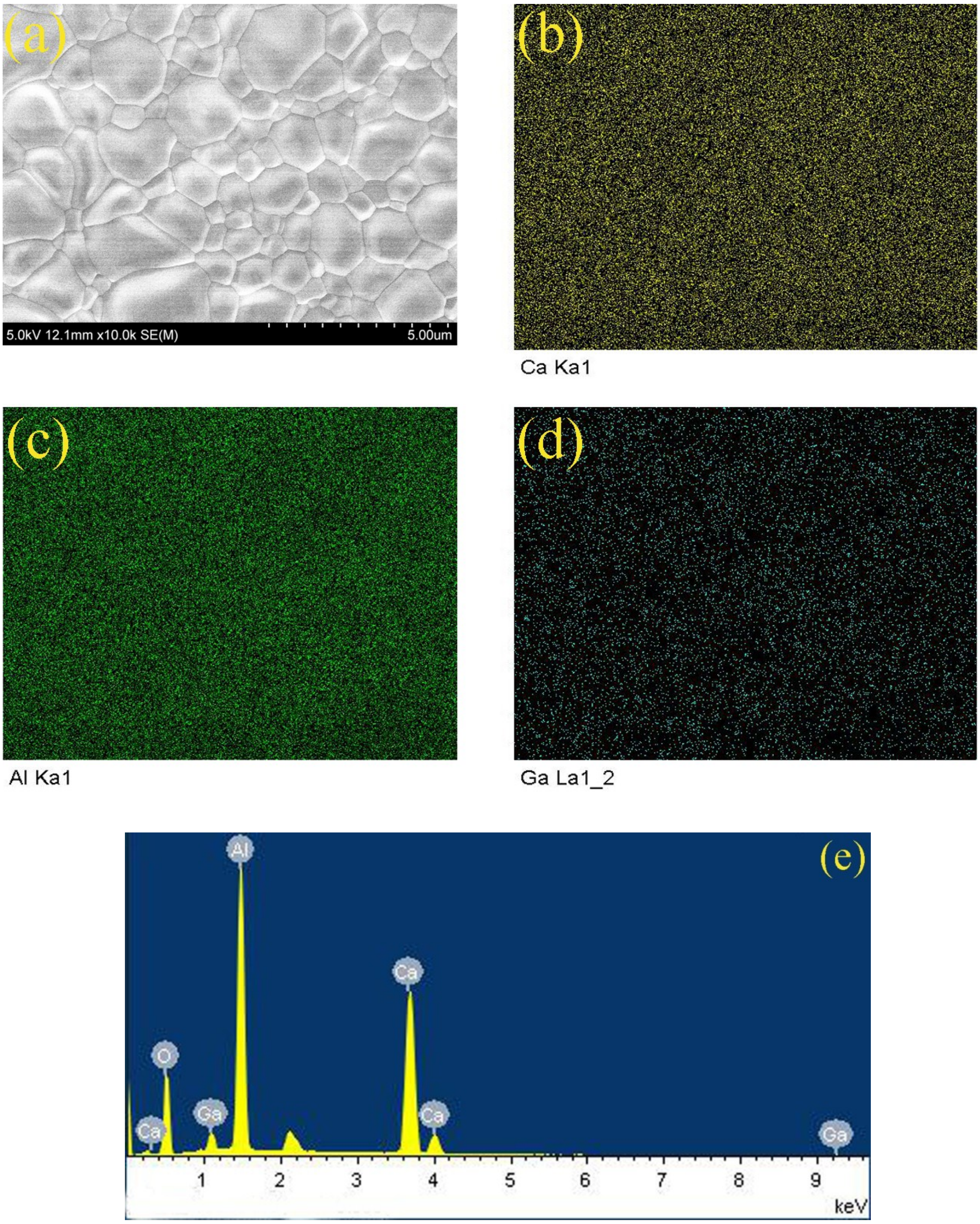

Figure S12. SEM micrograph of the composition $Ca_{12}Al_{13.0}Ga_{1.0}O_{33}$ (a), and EDS element distribution maps of Ca (b), Al (c), and Ga (d) ; picture (e) shows the element concentrations, the un-labeled peak is ascribed to the Au element that sprayed on the surface of the ceramic pellet before measurements. The relative element ratios for Ca : Al : Ga were revealed to be 12 : 13.11 : 0.92

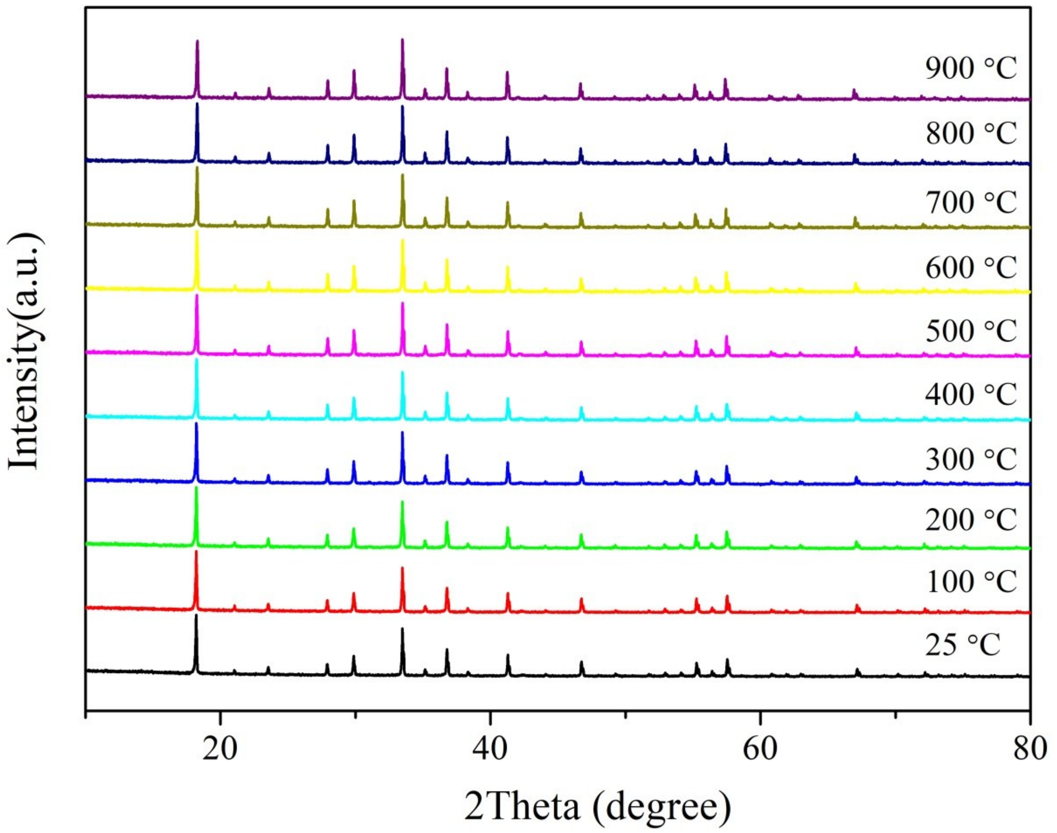

Figure S13. In-situ VT-XRD patterns of the sample x = 0.4
